# Microbiota of the Colonic Diverticula in the Complicated Form of Diverticulitis: A Case Report

**DOI:** 10.3390/life12122129

**Published:** 2022-12-16

**Authors:** Dina Yarullina, Yuliia Pankratova, Olga Karaseva, Tatiana Grigoryeva, Oleg Karpukhin

**Affiliations:** 1 Institute of Fundamental Medicine and Biology, Kazan Federal University, 420008 Kazan, Russia; 2 Department of Surgical Diseases, Kazan State Medical University, 420012 Kazan, Russia

**Keywords:** diverticulitis, inflamed diverticulum, non-inflamed diverticulum, microbiota

## Abstract

Intestinal microbiota appears to be implicated in the pathogenesis of diverticular disease. We present the case of a patient with diverticular colon disease complicated by a pelvic abscess. During the successful surgical treatment, two specimens were taken from the resected colon segment for the microbiota analysis: an inflamed and perforated diverticulum and a diverticulum without signs of inflammation. Culturing and 16S rRNA gene sequencing revealed significant changes in the microbial community structure and composition associated with the acute inflammation and perforation of the colonic diverticulum. The characteristics that are usually associated with the inflammatory process in the gut, namely reduced microbial diversity and richness, decreased *Firmicutes*-to-*Bacteroidetes* (F/B) ratio, depletion of butyrate-producing bacteria, and *Enterobacteriaceae* blooming, were more pronounced in the non-inflamed diverticulum rather than in the adjacent inflamed and perforated one. This is the first study of the intraluminal microbiota of the diverticular pockets, which is more relevant to the etiology of diverticular disease than mucosa-associated microbiota via biopsies and luminal microbiota via fecal samples.

## 1. Introduction

The incidence of diverticular disease (DD) is steadily increasing in the 21st century. In recent times, exacerbation of DD has become a frequent gastrointestinal complication of COVID-19 [1]. The clinical symptoms of DD arise as a result of inflammation in the intestinal mucosa of the diverticula, which are small outpouchings of the colon wall [2]. This inflammation may be induced by the local ischemia consequent of the compression of the vessels in the submucosal layer of the diverticula as well as microperforations of the diverticula walls injured by the dense fecal stones [3]. Changes in the intestinal microbiota composition may be implicated in the persistence of the inflammatory process and hence the progression of DD [4]. There have been few reports on the association between microbiota and diverticulosis with contradictory results [5]. The possible reason for the vagueness about the role of the intestinal microbiota in DD is that these studies were performed on patients with different disease severity (asymptomatic diverticulosis, Symptomatic Uncomplicated Diverticular Disease (SUDD), and diverticulitis or Segmental Colitis Associated with Diverticulosis (SCAD)) and assessed different kinds of microbiota (mucosa-associated microbiota via biopsies and luminal microbiota via fecal samples). Most of the studies declared no substantial differences in the gut microbiota composition between patients with diverticulosis and controls [6,7,8] except for non-significant changes in the relative abundance of some taxa: *Proteobacteria* and *Comamonadaceae* [7], *Akkermansia muciniphila* [8], and *Clostridium* cluster IV [9]. Interestingly, the mucosal microbiota was significantly different between “diseased” (affected by diverticulitis) and adjacent “healthy” tissue. The microbiota associated with a region of the sigmoid colon chronically affected by diverticulitis exhibited a relative overabundance of *Microbacteriaceae* and *Ascomycota* [10]. In patients with SUDD biopsies taken in the gut segment affected by diverticula, the biopsies were characterized by a higher abundance of *Enterobacteriaceae* and a lower abundance of *Bacteroides/Prevotella* group and *Akkermansia* compared with biopsies taken from the distant site. Notably, in control subjects without colonic diverticula and patients with asymptomatic diverticulosis, no differences between the mucosal microbiota of the diverticular or non-diverticular regions were detected [9]. These findings are evidence that we should consider diverticula as a unique niche that selectively promote the development of specific microbial communities, which may play a role in the progression of diverticulosis towards symptomatic forms [11]. The aim of this study is to characterize the juxta-mucosal microbiota in the lumens of diverticula from a patient who has undergone surgery for a pelvic abscess and diverticular perforation. Herein, using a conventional culture method and 16S rRNA-based sequencing approach, we assessed the microbiota composition of the inflamed perforated diverticulum (ID) and an adjacent non-inflamed diverticulum (NID) from the same colonic segment.

## 2. Case Report

A 52-year-old man was admitted at the Emergency Department of our hospital with complaints of pain in the lower abdomen, fever, chills, liquid stool 2–3 times per day, and general weakness over the previous week. The patient used rifaximin (400 mg orally twice daily) and metronidazole (500 mg orally three times daily) for self-medication but did not feel improvement. His medical history revealed an episode of acute diverticulitis with subsequent conservative treatment five years before this admission. The patient also had a history of psoriasis and hypertension (stage 2, risk 2).

**Figure 1:**
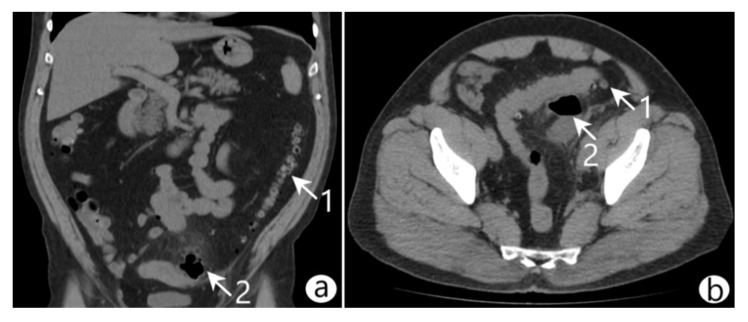
Coronal (**a**) and axial (**b**) abdominal CT scans revealed diverticulitis (arrow 1) of the sigmoid colon complicated by the formation of a pelvic abscess (arrow 2).

On admission, vital signs were as follows: body temperature 37.8 °C, blood pressure 110/70 mmHg, heart rate 86 beats/min, and respiratory rate 20/min. Laboratory blood tests showed the following: white blood cell (WBC) count 14.1 × 10^9^/L with neutrophilic granulocyte percentage 74.5%, red blood cell (RBC) count 4.3 × 10^12^/L, platelets 21.8 × 10^10^/L, hemoglobin (HGB) 11.2 g/dL, and hematocrit (HCT) 43.3%. The abdominal physical examination revealed symmetry, softness in palpation, no swelling, pain in the left side of the mesogastrium, and no symptoms of peritoneal irritation. The abdominal ultrasound indicated edema and thickening of the sigmoidal wall and a pericolic abscess. Computed tomography (CT) with intravenous contrast revealed an inflamed diverticulum in the sigmoid colon, a pelvic abscess (45 × 35 × 56 mm) containing gas and fluid, and infiltration of the surrounding adipose tissue (Figure 1). A diagnosis of diverticular colon disease complicated by the formation of a pelvic abscess (Hinchey II) was made.

The treatment strategy was determined according to the European Clinical Guidelines for Diverticular Disease [12]. Drainage of the abscess under ultrasound control and thus minimally invasive surgery was technically impossible due to the risk of bowel perforation since the intestine loops formed the walls of the abscess. The patient was offered surgical treatment, to which he gave consent. The preoperative examination revealed no contraindications. The preoperative preparation included bowel preparation, antibiotic prophylaxis, and prevention of venous thromboembolic complications.

**Figure 2:**
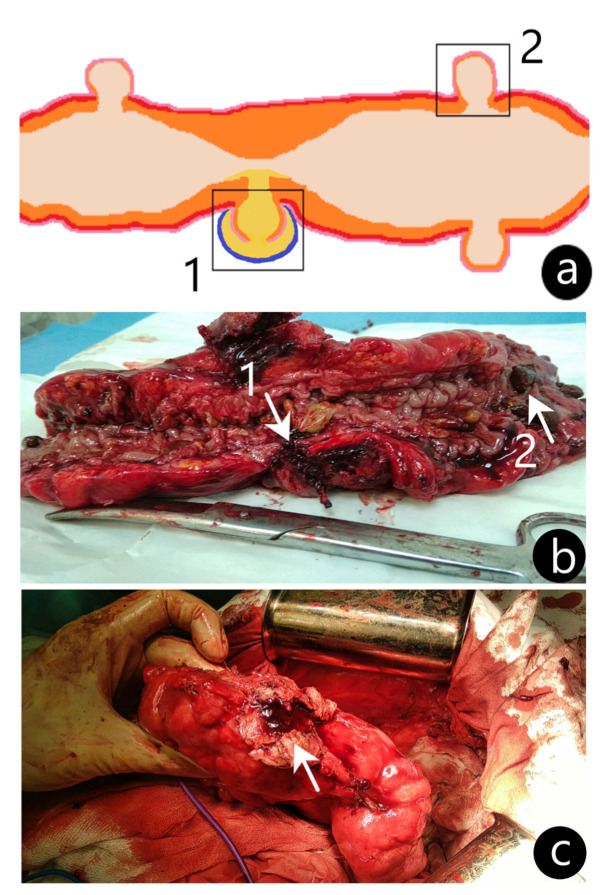
Visualization of the colonic specimens with diverticula. (**a**) An illustrated representation of the sigmoid colon with an inflamed perforated diverticulum (ID) (1) and non-inflamed diverticulum (NID) (2) obtained for analysis shown in boxes. (**b**) Postoperative image of the sigmoid colon resection with ID (arrow 1) and NID (arrow 2). (**c**) Intraoperative image of the sigmoid colon with capsule fragment of diverticular pericolic abscess (arrow).

The operation was performed by the laparotomic approach. The refusal of laparoscopic intervention was due to the presence of a dense inflammatory conglomerate consisting of the abdominal wall, sigmoid colon, omentum, and loops of the small intestine with a large (45 × 35 × 56 mm) pericolic abscess in the center. During mobilization, the pericolic abscess was opened, and the contents were taken for a bacteriological examination, which further revealed microbial cell counts of *Escherichia coli* as high as 10^3^ CFU/mL. A section of the sigmoid colon affected by the perforated diverticulum was resected; then, intestinal continuity was restored by an invaginating type of colorectal anastomosis. From the resected colon segment, two specimens were taken for the microbiota analysis: an inflamed and perforated diverticulum (ID) and a diverticulum without signs of inflammation (NID) (Figure 2). A routine histological examination of the specimens was performed in the Pathological Anatomy Department of the Republican Clinical Hospital (Kazan, Republic of Tatarstan). Both specimens were stained with hematoxylin and eosin and examined under the microscope (Zeiss Axio Scope A1, Goettingen, Germany). In the ID, a swollen intestinal wall with lymphocytic infiltration was revealed, while the NID showed no signs of acute inflammation.

We used a conventional culture method (Table 1) and 16S rRNA-based sequencing approach (Table 2) to assess the microbiota composition of both diverticula (for methods, see [13]). After trimming low-quality reads, the dereplicated reads were used for amplicon sequence variants (ASVs) inference. A total of 26,781 16S rRNA sequence reads was obtained following quality filtering, equating to 13,390 ± 3939 (mean ± SEM) reads per sample. After the removal of chimeras (1.75%) and non-bacterial sequences (9.38%), the number of mapped sequence reads per sample ranged from 8796 to 15,012 (mean ± SEM of 60,886 ± 4034). Following the removal of rare OTUs, defined as OTUs with 2 or less sequences across all the samples, rarefaction analysis demonstrated sufficient sequencing depth for a comparison analysis between the samples. The alpha-diversity analysis revealed an increased species richness and evenness in ID compared to NID (Table 2). These data were in opposition to the results of culturing, which showed nearly equal total bacterial growth (the culturable microbiota portion) in both diverticula (Table 1). The four phyla, *Firmicutes, Bacteroidetes, Proteobacteria*, and *Actinobacteria*, constituted the main bulk of microbiota in both diverticula, but in ID, the relative abundance of the *Firmicutes* phylum was significantly increased, and the *Proteobacteria* decreased as compared to NID (Table 3). The *Firmicutes*-to-*Bacteroidetes* (F/B) ratio is widely used to describe the gut microbiota composition. A decreased or an increased F/B ratio is regarded as an indicator of dysbiosis [14]. In ID, we found a F/B ratio of 69.3%/16.6%, which is significantly higher than in NID (*Firmicutes* 43.2%/*Bacteroidetes* 17.3%). Although the total load of bacteria of the *Bacteroidetes* phylum was similar between the two diverticula, we observed notable differences in the content of the two most abundant genera assigned to this phylum. In ID, we detected an increased abundance of *Bacteroides* (8%), while the *Prevotella* content was significantly depleted (5.4%). Conversely, NID showed an increase in the abundance of the genus *Prevotella* (9.6%) and a decrease in the abundance of the genus *Bacteroides* (5.4%) (Table 3).

We revealed a significant difference in the population of *Enterobacteriaceae* between the two samples (relative abundance of 5.4% in ID vs. 29.3% in NID) (Table 3). In this family, the 16S rRNA-based sequencing approach is characterized by the limited resolution at the genus and species levels [15]. We attempted to analyze enterobacteria using culture methods but obtained contradictory data regarding their amount in the two samples (Table 1), which may originate from the prevalence of uncultivated microorganisms in NID that could not be detected by the culture method.

Surprisingly, ID was characterized by the preponderant presence of the genera *Blautia, Coprococcus, Roseburia*, and *Faecalibacterium*, which are the butyrate-producing bacteria with anti-inflammatory properties. NID showed an increase in the abundance of the genera *Streptococcus* and *Ralstonia* and a decrease in the abundance of *Collinsella* as compared with ID (Table 3).

The postoperative period proceeded without complications, and the patient was discharged on the sixth day after surgical treatment to a rehabilitation facility. Six months later, he was seen in the clinic and reported no further clinical symptoms of diverticulitis; laboratory tests and control sonography results revealed no signs of the disease.

## 3. Discussion

Several lines of evidence suggest a role for intestinal microbiota in the pathogenesis of DD. Substantial changes in the gut microbial community structure have been detected in the patients suffering from DD [4,5]. Probiotics are able to restore changes in microbiota composition and, therefore, are potentially applicable in the treatment of this disease [16]. The beneficial effects of rifaximin administration in SUDD also support the involvement of bacteria in the pathophysiological process [17]. In line with the possible etiological role of microbiota in diverticulitis, we assume that disease-causing infectious agents might be enriched at sites of active inflammation relative to comparatively unaffected mucosa. We have, therefore, used a conventional culture-based method and bacterial 16S rRNA gene sequencing technology to compare the mucosa-associated microbiota from ID and NID from the same colonic segment. It has repeatedly been shown that DD generally affects only limited segments of the colon [11], and thus diverticular pockets are more relevant samples than feces or mucosal biopsies.

The microbiota profiles differed substantially between ID and adjacent NID. Inflammation and perforation of the diverticulum were accompanied by an increased phylogenetic diversity and total amount of bacteria, an abundance of *Firmicutes,* a depletion of *Proteobacteria*, and an advanced F/B ratio in the gut microbiota as compared to NID. The most drastic difference between the two samples was in reference to the overabundance of uncultivated *Enterobacteriaceae* family in NID, as follows from the results of sequencing and culture methods. Different from our study, it has been well documented that intestinal inflammation is usually associated with a reduction in microbial richness and diversity, decreased F/B ratio, and increased abundance of potential proinflammatory *Proteobacteria* [18]. Fecal microbiota in diverticulosis and SUDD was reported to be deficient in *Clostridium* cluster IV, which is now classified as several genera in the family *Ruminococcaceae*, including the anti-inflammatory and butyrate-producing species *Faecalibacterium prausnitzii* [9]. In contrast, ID in our study preponderated over NID in the relative abundance of *Ruminococcaceae* and *F. prausnitzii* in 1.6 and 1.9 times, respectively. The other butyrate producers, such as *Blautia, Coprococcus*, *Roseburia,* and *Faecalibacterium*, were significantly increased in ID as compared to NID. Overall, the characteristics that are usually associated with the inflammatory process in the gut, namely reduced microbial diversity and richness, decreased F/B ratio, depletion of butyrate-producing bacteria, and *Enterobacteriaceae* blooming, were more pronounced in NID rather than in ID. Of these two studied samples, the microbiota of NID corresponded to the literature data on the fecal or mucosal microbiota of patients with SUDD [9] or acute diverticulitis [19].

We found an inverse relationship in enrichment between *Prevotella* and *Bacteroides* in both ID and NID. The *Prevotella*-to-*Bacteroides* ratios (P/B ratios) in the studied diverticula were 0.67 and 1.79, respectively. These two genera of the *Bacteroidales* order have previously been suggested as the main determinants of two of the three enterotypes. Enterotype 1 is characterized by a relatively high abundance of *Bacteroides* spp., enterotype 2 by *Prevotella* spp., and enterotype 3 by *Ruminococcus* [20]. The understanding of the biological significance of enterotypes is still limited. Enterotype 1 has previously been associated with the Western diet that has a high intake of animal protein and saturated fat, whereas enterotype 2 has been reported to be predominant in individuals that consume carbohydrate- and fiber-rich diets [21]. Nevertheless, individuals belonging to enterotype 2 had significantly higher plasma concentrations of cholesterol and trimethylamine-*N*-oxide (TMAO), a proatherogenic metabolite, than individuals belonging to enterotype 1 [22,23]. Our data suggest that inflammation in the diverticula is associated with a shift in the P/B ratio towards a predominance of *Bacteroides.*

The other taxa whose abundance differed significantly in ID and NID were *Collinsella aerofaciens, Streptococcus luteciae,* and *Ralstonia* spp. *C. aerofaciens* was increased in ID compared to NID (2.3 vs. 0.4%, respectively), while two other taxa were preponderant in NID compared to ID (5.5 vs. 1.4% and 4.3 vs. 1.0%, respectively). Although the association of these known intestinal commensals with some diseases has been reported [24,25,26], the mechanism of their probable pathophysiological activity remains obscure.

Thus, our results indicate several pronounced changes in the intraluminal mucosal microbiota of the colonic diverticulum injured by acute inflammation and perforation. This pilot study presents only a snapshot of the intestinal microbiota within the diverticula confined space of which provides persistence of normal and pathogenic microbiota. Observed changes in the microbiota may reflect location-associated differences and warrant further microbiota studies throughout the length of the colon. Another important limitation of this study is that gut microbiota varies greatly between individuals, and the results obtained for one patient may not be widely generalizable. Further studies should help to elucidate the role of microbiota in the pathogenesis of DD and its progression towards more severe forms. The prediction of diverticulitis risks based on microbiota biomarkers opens the perspective of prophylaxis of disease relapses, more timely treatment, and prevention of complications, including the deliberate use of antibiotics for diverticulitis treatment and application of probiotics and fecal microbiota transplantation (FMT) for specific modulation of intestinal microbiota.

## Figures and Tables

**Table 1:** Microbiota of diverticula assessed by culture method.

Groups of Microorganisms, lg CFU/g	Inflamed Diverticulum (ID)	Non-Inflamed Diverticulum (NID)
Total bacterial growth of aerobic bacteria	13.45	13.37
Total bacterial growth of anaerobic bacteria	11.90	12.18
Lactobacilli	12.15	12.29
*Bifidobacterium* spp.	11.99	nd
*Enterobacteriaceae*	12.27	7.81
*Salmonella* spp., *Shigella* spp.	nd	nd

nd—not determined.

**Table 2:** Number of reads and alpha-diversity indices of the diverticula.

	Inflamed Diverticulum (ID)	Non-Inflamed Diverticulum (NID)
Raw reads	16,176	10,605
Non-chimeric reads	15,765	10,587
Reads with OTU	15,012	8796
Alpha-diversity indices		
Observed OTUs	665	458
Phylogenetic diversity	41.0	32.6
Chao1	670.4	496.6
Shannon	7.3	6.1
Simpson	0.98	0.92

**Table 3:** Major taxa detected in diverticula by 16S ribosomal RNA gene sequencing *.

Taxonomy	Relative Abundance, %	
	Inflamed Diverticulum (ID)	Non-Inflamed Diverticulum (NID)
*Actinobacteria*	3.6	3.2
* Actinomycetales*	0.3	2.0
* Coriobacteriales*	3.3	0.6
* Coriobacteriaceae*	3.3	0.6
* Collinsella*	2.3	0.4
*Bacteroidetes*	16.6	17.3
* Bacteroidales*	16.6	17.3
* Bacteroidaceae*	8.0	5.4
* Bacteroides*	8.0	5.4
* Prevotellaceae*	5.4	9.6
* Prevotella*	5.4	9.6
*Firmicutes*	69.3	43.2
* Lactobacillales*	2.5	5.8
* Streptococcaceae*	1.6	5.5
* Streptococcus*	1.4	5.5
* Clostridiales*	66.3	36.2
* Lachnospiraceae*	29.0	14.8
* Blautia*	8.7	4.4
* Coprococcus*	4.7	1.4
* Roseburia*	4.7	1.4
* Ruminococcaceae*	23.2	14.3
* Faecalibacterium*	9.2	4.9
* Ruminococcus*	3.0	2.1
Unknown *Ruminococcaceae*	9.9	7.0
*Proteobacteria*	9.5	36.0
* Burkholderiales*	1.3	6.3
* Oxalobacteraceae*	1.0	4.3
* Ralstonia*	1.0	4.3
* Enterobacteriales*	5.4	29.3
* Enterobacteriaceae*	5.4	29.3
Unknown *Enterobacteriaceae*	5.4	29.3
* Pseudomonadales*	1.4	0.0
Other phyla	1	0.3

* See Table S1 for the complete list of taxa detected in the diverticula by 16S rRNA gene sequencing.

## Data Availability

The datasets generated and/or analyzed during the current study are available from the corresponding author upon reasonable request.

## Supplementary Materials

The following supporting information can be downloaded at: https://www.mdpi.com/article/10.3390/life12122129/s1, Table S1: Taxonomic profiling of the diverticula by 16S ribosomal RNA gene sequencing.
